# Can the radiomics features of intracranial aneurysms predict the prognosis of aneurysmal subarachnoid hemorrhage?

**DOI:** 10.3389/fnins.2024.1446784

**Published:** 2024-10-21

**Authors:** Tianxing Huang, Wenjie Li, Yu Zhou, Weijia Zhong, Zhiming Zhou

**Affiliations:** ^1^Department of Radiology, The Second Affiliated Hospital of Chongqing Medical University, Chongqing, China; ^2^Department of Radiology, People’s Hospital of Linshui County, Guang’an, China

**Keywords:** ruptured intracranial aneurysm, radiomics features, aneurysmal subarachnoid hemorrhage, prognosis, model construction

## Abstract

**Objectives:**

This study attempted to determine potential predictors among radiomics features for poor prognosis in aneurysmal subarachnoid hemorrhage (aSAH), develop models for prediction, and verify their predictive power.

**Methods:**

In total, 252 patients with aSAH were included in this study and categorized into favorable and poor outcome groups based on the modified Rankin Scale score 3 months after event. Radiomics features of the ruptured intracranial aneurysm extracted from computed tomography angiography images were selected using least absolute shrinkage and selection operator regression and 10-fold cross-validation. A radiomics score was created by selecting the optimal features. Other risk factors for a poor prognosis were screened using multivariate regression analysis. Three models (clinical, aneurysm, and clinical-aneurysm combined models) were developed. The performance of the models was assessed using receiver operating characteristic (ROC) curves. A clinical-aneurysm combined nomogram was constructed to forecast the risk of poor prognosis in patients with aSAH.

**Results:**

A total of three clinical variables and six radiomics features were shown to have a significant association with poor prognosis in patients with aSAH. In the training cohort, the clinical, aneurysm, and clinical-aneurysm combined models had areas under the ROC curves of 0.846, 0.762, and 0.893, respectively. In the testing cohort, these models had areas under the ROC curves of 0.848, 0.753, and 0.869, respectively.

**Conclusion:**

The radiomics characteristics of ruptured intracranial aneurysms are valuable to predict prognosis after aSAH. The clinical-aneurysm combined model exhibited the best among the three models. The clinical-aneurysm combined nomogram is a reliable and effective tool for predicting poor prognosis in patients with aSAH.

## 1 Introduction

Aneurysmal subarachnoid hemorrhage (aSAH) is a highly lethal and debilitating kind of hemorrhagic stroke (Virani et al., 2020; Connolly et al., 2012). Approximately 33% of survivors exhibit significant impairments and rely on others for their daily functioning (Rinkel and Algra, 2011; Mackey et al., 2016; Petridis et al., 2017). Therefore, an early and reliable predictive model to evaluate the prognosis of aSAH is urgently required.

Previous studies (Lagares et al., 2015; van der Steen et al., 2020) have shown that the total volume of the subarachnoid hemorrhage (SAH) is closely related to the prognosis of aSAH. The Fisher scale is widely used in clinical practice to estimate the severity of blood loss in subarachnoid hemorrhage (Woo et al., 2017). However, this scale is a crude assessment of blood loss and has limited predictive ability. Some studies (van der Steen et al., 2020; Jiménez-Roldán et al., 2013) in which the traditional Fisher scale was replaced with the total subarachnoid hemorrhage volume to predict the prognosis of aSAH reported good predictive power. The inclusion of more comprehensive factors, such as aneurysm characteristics (size, shape, and surface irregularities) (Duan et al., 2018; Zheng et al., 2016; Wiebers et al., 2003) influencing the extent and distribution of subarachnoid hemorrhage and consequently the degree of brain injury may help improve predictive ability (Parekh et al., 2023; Duan et al., 2016).

Recently, radiomics has become widely used in radiological studies. Being a high-throughput technology, it efficiently extracts a large number of features from images and offers further information for the purposes of diagnosis, prognosis evaluation, and treatment response assessment (Lambin et al., 2012). More comprehensive image features can be obtained using radiomics. Studies using radiomics to extract additional aneurysm-related features have achieved good results in predicting aneurysm rupture (Liu et al., 2019; Zhu et al., 2021). However, these features cannot be used to predict the prognosis of aSAH, and their predictive performance remains unknown.

The aim of this research was to find independent factors among radiomics features for the prediction of aSAH prognosis. Three different models (clinical, aneurysm, and clinical-ancurysm (C-A) combined) were created based on the results, and their predictive power was evaluated to determine which one was more appropriate to help clinicians to predict the prognosis of patients affected by aSAH.

## 2 Methods

### 2.1 Participants

This retrospective study received approval from the Human Experimentation Ethical Standards Committee of the Second Affiliated Hospital of the Chongqing Medical University. We conducted a retrospective analysis of 312 patients, all of whom were above the age of 18 and has been diagnosed with SAH using non-contrast computed tomography (NCCT). The data was collected from two centers in our hospital, from January 2019 to November 2023. The criteria for exclusion were as follows: (a) SAH caused by non-aneurysmal causes (such as trauma, hypertensive cerebral hemorrhage, or vascular malformation). (b) History of intracranial tumors or stroke with neurological dysfunction. (c) Poor-quality computed tomography angiography (CTA) and NCCT images (d) History of intracranial aneurysm surgery. (e) Incomplete clinical data.

### 2.2 Prognosis evaluation

The clinical functional outcome of patients with aSAH was evaluated using the modified Rankin Scale (mRS) at 90 days after onset, either by structured telephone interviews or by reviewing medical records. Patients with a score of 4–6 range were considered to exhibit a poor outcome, while those with scores within the 0–3 range were considered to manifest a favorable outcome (Hanley et al., 2017).

### 2.3 Demographic and clinical date

We collected the demographic data and clinical characteristics of each participant from the information system of our institution. The demographic data encompassed age, gender, medical history pertaining to coronary heart disease, hypertension, and diabetes mellitus; smoking history, and alcohol intake. Clinical features included Hunt–Hess score on admission, intracranial infection, acute hydrocephalus during hospitalization, and laboratory examinations before surgical intervention. Laboratory examinations included white blood cell count, neutrophil count, lymphocyte count, neutrophil-to-lymphocyte ratio, platelet count, and hemoglobin count. To obtain the Hunt–Hess score, we followed the protocol described in previous studies (Konczalla et al., 2018).

### 2.4 Image acquisition and analysis

CT of the head was conducted utilizing a multi-slice spiral CT scanner (Siemens Healthcare, Somatom Definition Force, Canon Medical Systems, or Aquilion ONE). The procedures for CT and CTA were as follows: 200–300 mA, 110–120 kV, layer thickness of 1 mm, and a 512 × 512 matrix. The administered amount of the contrast agent (iohexol solution) ranged from 150 to 300 mg/kg, and the rate of injection was 4.5–5.0 mL/s.

To reduce the impact of potential differences between the CT scanners on the image before image analysis, we performed image pre-processing: (a) the CTA data were resampled to 1.0 × 1.0 × 1.0 mm^3^ voxel size; (b) gray level discretization were resampled on a fixed number of 256 bins; (c) the CTA images were set to a window level of 50 Hounsfield units (Hu) and a window width of 110 Hu.

The following information was obtained from the NCCT images before the surgical intervention: volume of subarachnoid hemorrhage, mean CT value of subarachnoid hemorrhage, midline shift (MLS), intracerebral hemorrhage (ICH), and intraventricular hemorrhage (IVH). The volume and mean CT value of subarachnoid hemorrhage were measured using StrokeDoc software,^1^ which started with automatic segmentation of different brain structures and identification of the volume and CT value of blood; all segmentations were checked and corrected by an experienced radiologist who was blinded to all clinical data and outcomes. A midline shift >4 mm was considered an MLS (Yang et al., 2018).

### 2.5 Radiomics analysis

**Figure 1:**
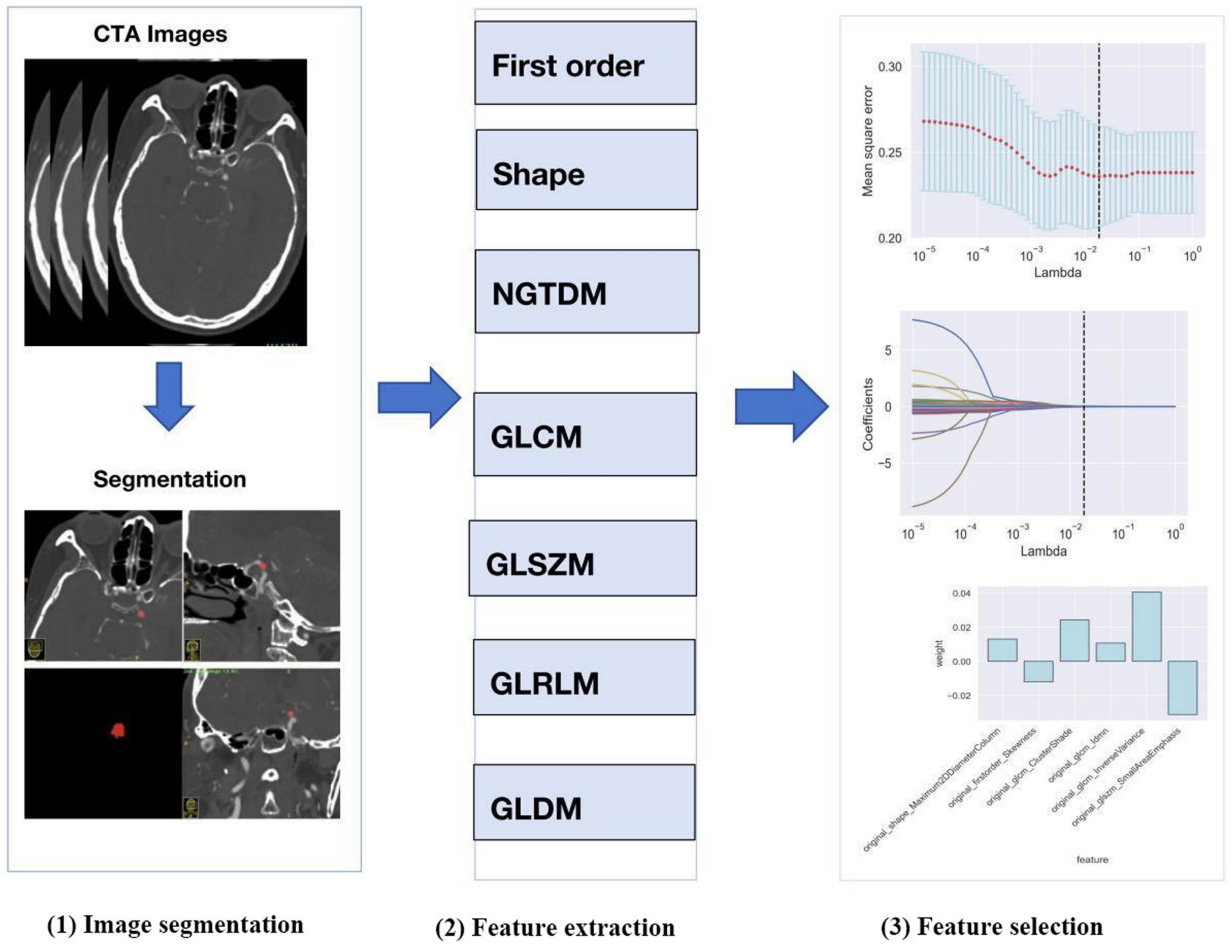
Workflow of radiomics analysis.

The process of radiomics analysis is illustrated in Figure 1, which consists of image segmentation, feature extraction, and feature selection.

#### 2.5.1 Image segmentation

We selected the region of interest (ROI) of the ruptured intracranial aneurysm (IA) from CTA images obtained before the surgical intervention. When multiple aneurysms were found, surgical records were used to identify the aneurysm responsible for the hemorrhage. The ROI of each IA was generated using the ITK-SNAP software package.^2^ ROIs were manually designated along the boundary of the aneurysm on each slice of the original axial, reconstructed coronal and sagittal images.

#### 2.5.2 Reproducibility analysis

Data from 50 randomly selected cases in the training cohort was used for inter-observer and intra-observer reproducibility assessment. The ROIs for each patient were semi-automatically segmented again by the same radiologist after 7 days and by a different trained radiologist using the same method. The interclass correlation coefficient (ICC) was employed for assessment of inter-observer and intra-observer agreement.

#### 2.5.3 Feature extraction

Following the segmentation of ROIs, the radiomics features were extracted automatically using Python software (version 3.2) from eight feature groups. These groups included the neighborhood gray level dependency matrix, the neighborhood gray difference matrix, gray level run length matrix, the gray level size zone matrix, the gray level co-occurrence matrix, 2D shape, 3D shape, and first-order statistics. A total of 107 radiomics features were acquired from each IA.

#### 2.5.4 Feature selection

Python (version 3.8.1) was used to select the radiomics features. A *t*-test was used to reduce the dimensionality of the radiomics features, and those exhibiting *p* < 0.05 in the test were retained. The least absolute shrinkage and selection operator (LASSO) was then employed to select the optimal subset of features from the remaining set, while parameter tuning performed with 10-fold cross-validation to mitigate overfitting.

### 2.6 Model construction

In the training cohort, independent risk factors for a poor prognosis of aSAH were examined using univariate and multivariate logistic regression analyses (*p* < 0.05). We utilized statistically significant independent clinical features (*p* < 0.05) to construct a clinical model by logistic regression analysis.

The radiomics score (R-score) for each patient was used to construct the aneurysm model. R-score of each patient was calculated according to the following formula based on the optimal features: R-score = (∑β_i_*x_i_) + *β*_0_, where *x_i_* represents the *i*th feature, *β_i_* represents the correlation coefficient in LASSO, and *β*_0_ represents the constant.

Finally, the C-A combined model, which integrates independent clinical risk factors and R-scores, was developed using logistic regression. We conducted independent verification of these models in the test cohort and assessed their performance by computing the area under the curve (AUC).

### 2.7 Model evaluation

We employed the receiver operating characteristic (ROC) curve to assess the effectiveness of each model and computed its specificity, sensitivity, accuracy, and AUC. The DeLong test was employed to compare the AUCs of the various models. Calibration and decision curve analysis (DCA) were employed to evaluate the calibration ability and clinical usefulness of the C-A combined model, respectively.

### 2.8 Statistical analysis

The statistical analysis was conducted using R software (version 3.6.0). Variables were characterized using measures such as mean ± standard deviation, median (interquartile range), or counts (percentages). The Kolmogorov–Smirnov test was used to assess the normality of the continuous variables. The Mann–Whitney *U* test, student’s *t*-test or Fisher’s exact test were employed to ascertain differences between groups. A *p*-value less than 0.05 was deemed to be statistically significant.

## 3 Results

### 3.1 Demographic and clinical characteristics

This study included 252 patients with aSAH, with 171 and 81 patients in the training and testing cohorts, respectively. With the exceptions of neutrophil count (*p* = 0.032) and ICH (*p* = 0.045), there were no significant differences in the demographic, clinical, and CT imaging characteristics between the training and testing cohorts (Table 1). In the training cohort, patients were divided into a favorable outcome group (*n* = 105) or the poor outcome group (*n* = 66) based on the mRS. Univariate analysis (Table 2) of the training cohorts revealed that in terms of demographic data and medical history, the incidence of hypertension, coronary heart disease, and diabetes was lower in the group with favorable outcome. Age (*p* < 0.001), Hunt–Hess score at admission (*p* = 0.002), hemoglobin count (*p* < 0.001), subarachnoid hemorrhage volume (*p* < 0.001), ICH (*p* < 0.001), and IVH (*p* < 0.001) were risk factors associated with poor outcomes after 90-days.

**Table 1:** Patients’ characteristics in the training and testing cohorts.

Variables	Training cohort (*n* = 171)	Testing cohort (*n* = 81)	*p*-value
Male, *n* (%)	76 (44.4%)	34 (42.0%)	0.816
Age, mean (SD)	57.74 (11.59)	56.42 (12.24)	0.409
Hypertension, *n* (%)	90 (52.6%)	37 (45.7%)	0.370
Coronary heart disease, *n* (%)	4 (2.3%)	0 (0.0%)	0.309
Diabetes, *n* (%)	8 (4.7%)	3 (3.7%)	0.981
Smoking history, *n* (%)	23 (13.5%)	8 (9.9%)	0.548
Drinking history, *n* (%)	19 (11.1%)	7 (8.6%)	0.568
Hunt–Hess score on admission, median (IQR)	2.00 (2.00, 2.50)	2.00 (2.00, 2.00)	0.832
Acute hydrocephalus, *n* (%)	7 (4.1%)	7 (8.6%)	0.239
Intracranial infection, *n* (%)	4 (2.3%)	2 (2.5%)	>0.999
WBC count, mean (SD)	12.74 (4.31)	13.83 (6.08)	0.103
Neutrophil count, mean (SD)	11.20 (4.13)	13.19 (10.49)	0.032
Lymphocyte count, median (IQR)	0.86 (0.65, 1.12)	0.91 (0.68, 1.17)	0.600
NLR, mean (SD)	13.91 (7.43)	16.12 (16.39)	0.141
Hemoglobin count, mean (SD)	124.26 (20.98)	120.22 (29.21)	0.212
PLT count, mean (SD)	206.16 (61.10)	209.65 (56.64)	0.665
Midline shift, n (%)	6 (3.5%)	3 (3.7%)	>0.999
Volume of SAH, median (IQR)	17.24 (8.20, 29.82)	17.96 (8.50, 27.48)	0.745
CT value of SAH, median (IQR)	57.00 (54.00, 60.00)	56.00 (54.00, 59.00)	0.494
ICH, *n* (%)	33 (19.3%)	22 (27.2%)	0.212
IVH, *n* (%)	83 (48.5%)	51 (63.0%)	0.045
R-score, mean (SD)	38.02 (6.96)	38.12 (9.72)	0.929
Poor prognosis, *n* (%)	66 (38.6%)	30 (37.0%)	0.812

PLT, platelet count; WBC, white blood cell count; NLR, neutrophil to lymphocyte ratio; aSAH, aneurysmal subarachnoid hemorrhage; R-score, radiomics score; ICH, intracerebral hemorrhage; IVH, intraventricular hemorrhage.

**Table 2:** Comparison of baseline characteristics of patients with aSAH in poor outcome group and favorable outcome group.

Variables	Favorable outcome group (*n* = 105)	Poor outcome group (*n* = 66)	*p*-value
Male, *n* (%)	53 (50.5%)	23 (34.8%)	0.065
Age, mean (SD)	54.31 (9.79)	63.18 (12.19)	<0.001
Hypertension, *n* (%)	52 (49.5%)	29 (43.9%)	0.579
Coronary heart disease, *n* (%)	1 (1.0%)	3 (4.5%)	0.300
Diabetes, *n* (%)	3 (2.9%)	5 (7.6%)	0.293
Smoking history, *n* (%)	15 (14.3%)	8 (12.1%)	0.862
Drinking history, *n* (%)	13 (12.4%)	6 (9.1%)	0.345
Hunt–Hess score on admission, median (IQR)	2.00 (2.00, 2.00)	2.00 (2.00, 3.00)	0.002
Acute hydrocephalus, *n* (%)	2 (1.9%)	5 (7.6%)	0.109
Intracranial infection, *n* (%)	1 (1%)	3 (4.5%)	0.300
WBC count, mean (SD)	12.55 (3.59)	13.05 (5.28)	0.464
Neutrophil count, mean (SD)	11.09 (3.55)	11.38 (4.94)	0.653
Lymphocyte count, median (IQR)	0.88 (0.65, 1.11)	0.84 (0.63, 1.14)	0.851
NLR, mean (SD)	13.93 (7.07)	13.87 (8.02)	0.955
Hemoglobin count, mean (SD)	128.88 (18.13)	116.90 (23.15)	<0.001
PLT count, mean (SD)	204.79 (58.25)	208.35 (65.79)	0.712
Midline shift, n (%)	3 (2.9%)	3 (4.5%)	0.677
Volume of SAH, median (IQR)	12.50 (5.40, 24.60)	25.35 (16.69, 35.12)	<0.001
CT value of SAH, median (IQR)	56.00 (53.00, 60.00)	57.00 (56.00, 60.00)	0.134
ICH, *n* (%)	5 (4.8%)	28 (42.4%)	<0.001
IVH, *n* (%)	37 (35.2%)	46 (69.7%)	<0.001
R-score, mean (SD)	35.50 (6.20%)	42.03 (6.23)	<0.001

PLT, platelet count; WBC, white blood cell count; NLR, neutrophil to lymphocyte ratio; aSAH, aneurysmal subarachnoid hemorrhage; R-score, radiomics score; ICH, intracerebral hemorrhage; IVH, intraventricular hemorrhage.

Multiple regression (Table 3) showed that age [odds ratio (OR), 1.061; 95% CI, 1.010–1.115; *p* = 0.018], the volume of SAH (OR, 1.032; 95% CI, 1.003–1.063; *p* = 0.031), ICH (OR, 0.132; 95% CI, 0.036–0.483; *p* = 0.002) and R-score (OR, 1.21;95% CI, 1.107–1.322; *p* < 0.001) were independent risk predictors of poor outcomes in the training cohort.

**Table 3:** Multivariate analysis of baseline characteristics in the training cohort.

Variables	OR	95% CI	*p*-value
Male	0.655	0.245–1.751	0.399
Age	1.061	1.01–1.115	0.018
Hunt–Hess score on admission	1.034	0.504–2.121	0.927
Hemoglobin count	0.984	0.961–1.008	0.192
The volume of SAH	1.032	1.003–1.063	0.031
R-score	1.21	1.107–1.322	<0.001
ICH	0.132	0.036–0.483	0.002
IVH	0.493	0.192–1.267	0.142

OR, odds ratio; CI, confidence interval; aSAH, aneurysmal subarachnoid hemorrhage; R-score, radiomics score; ICH, intracerebral hemorrhage; IVH, intraventricular hemorrhage.

### 3.2 Radiomics analysis

The results of the intra-observer reproducibility test showed that 97 radiomics features (90.7%) had ICCs >0.8, and the results of the inter-observer reproducibility test demonstrated that 100 radiomics features (93.5%) had ICCs >0.8.

**Figure 2:**
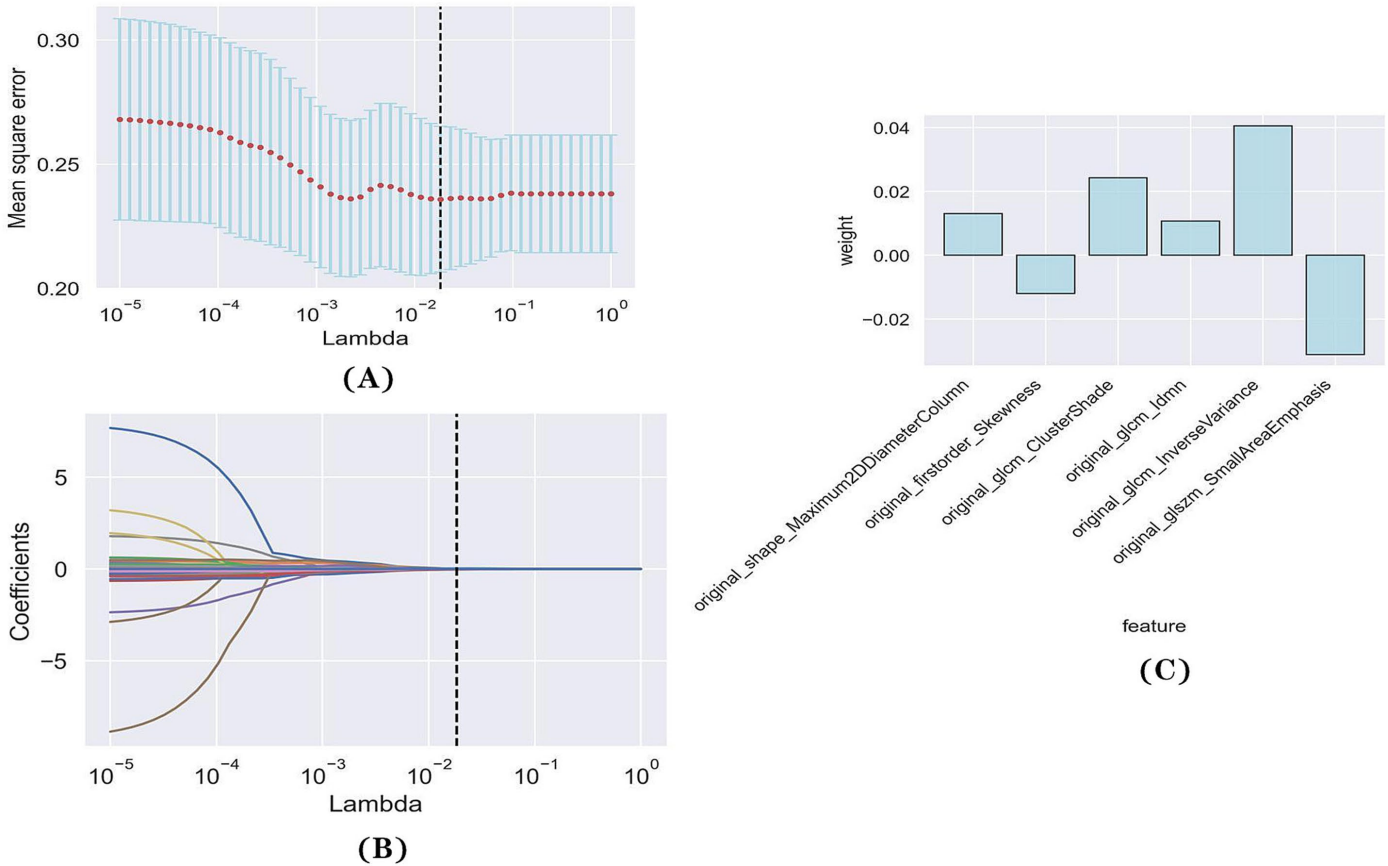
Feature selection with LASSO regression. **(A)** Tuning parameter (lambda) selection in the LASSO regression using 10-fold cross-validation. **(B)** LASSO regression coefficient analysis of the 39 radiomics features. Each coloured line represents the coefficient of each feature. **(C)** The *y*-axis represents individual radiomics features, with their coefficients in the LASSO regression analysis are plotted on the *x*-axis.

Of the 107 radiomics features, only 39 features with significant differences between the favorable and poor prognostic groups were retained (*t*-test, *p* < 0.05). Following LASSO and 10-fold cross-validation, six features were acquired (Figures 2A,B). The values of the coefficients for each feature are displayed in Figure 2C.

### 3.3 Model evaluation

**Figure 3:**
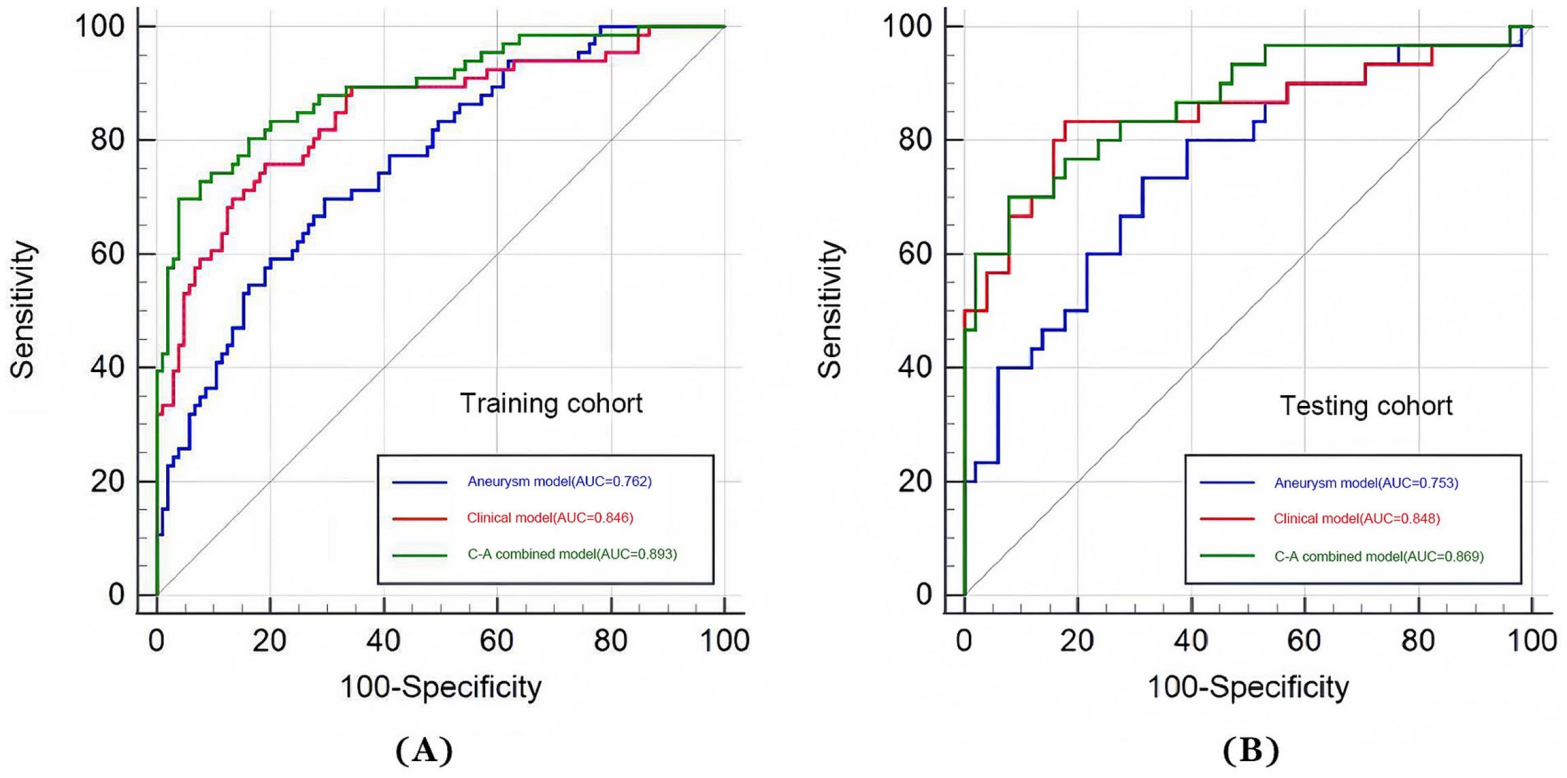
ROC curves of three models (aneurysm, clinical, and C-A combined model). **(A)** The ROC curves of three models for predicting the risk of poor prognosis of aSAH in the training cohort. **(B)** The ROC curves of three models for predicting the risk of poor prognosis of aSAH in the testing cohort.

The ROC curves of the three models in the training and testing cohorts for predicting a poor prognosis of aSAH are shown in Figures 3A,B. The aneurysm model had an AUC of 0.762 and 0.753 for the training and testing cohorts, respectively. The AUCs of the clinical model based on age and the volumes of SAH and ICH were 0.846 and 0.848 in the training and testing cohorts, respectively. The AUCs of the C-A combined model in the training and testing cohorts were 0.893and 0.869, respectively, showing the best predictive performance among the three models. In the testing cohort, DeLong tests showed that there were no statistical differences between the C-A combined model and the clinical model, or between the clinical model and the aneurysm model, whereas there was a significant difference between the C-A combined model and the Aneurysm model (*p* = 0.0087). The performance metrics of the three models, including sensitivity, specificity, positive predictive value, negative predictive value, and accuracy in testing cohorts are shown in Table 4.

**Table 4:** The performance metrics of the three models in testing cohorts.

Models	Sensitivity	Specificity	Positive predictive value	Negative predictive value	Accuracy
Clinical model	83.33 [65.3–94.4]	82.35 [69.1–91.6]	73.5 [60.6–83.7]	89.4 [78.9–95.0]	82.7 [72.9–89.5]
Aneurysm model	73.33 [54.1–87.7]	68.63 [54.1–80.9]	57.9 [46.5–68.5]	81.4 [70.1–89.1]	70.4 [59.7–79.3]
C-A combined model	70.00 [50.6–85.3]	92.16 [81.1–97.8]	84.0 [66.6–93.3]	83.9 [75.0–90.1]	84.0 [74.3–90.5]

**Figure 4:**
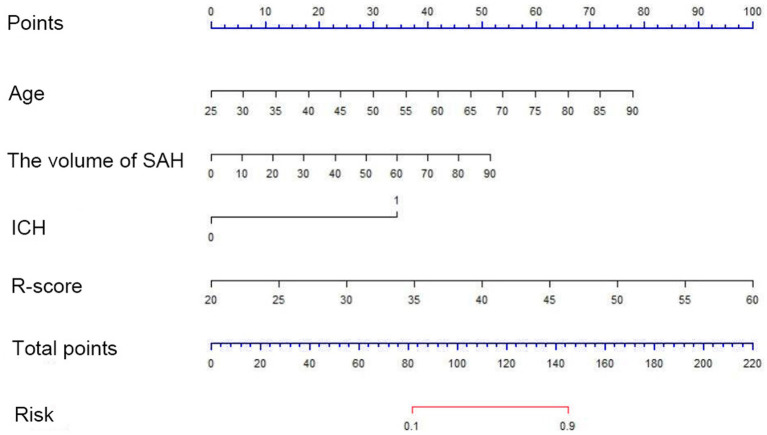
A C-A nomogram for assessing the risk of poor prognosis of aSAH. The nomogram is used by first summing the points corresponding to all predictors and then find the corresponding risk of poor prognosis of aSAH.

**Figure 5:**
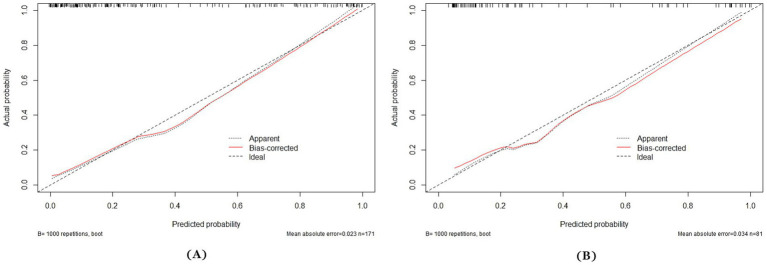
Calibration curves of the C-A combined model. **(A)** The calibration curve of the C-A combined model for predicting the risk of poor prognosis of aSAH in the training cohort. **(B)** The calibration curve of the C-A combined model for predicting the risk of poor prognosis of aSAH in the testing cohort.

**Figure 6:**
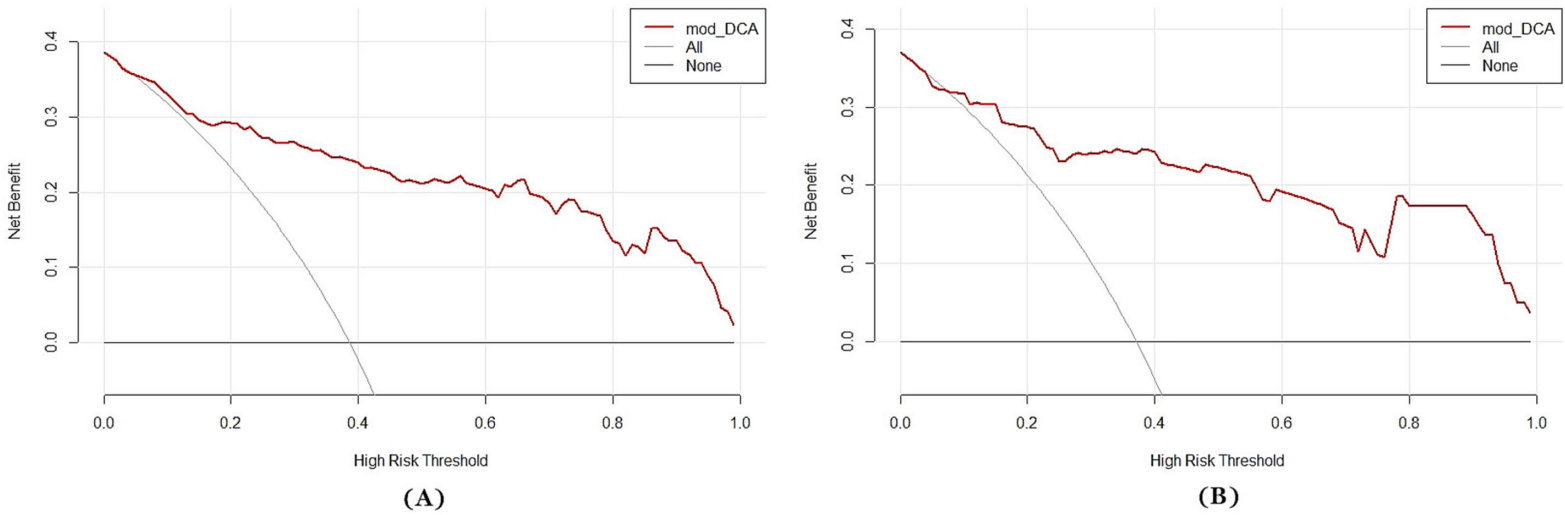
Decision curves of the C-A combined model. **(A)** The decision curve of the C-A combined model for predicting the risk of poor prognosis of aSAH in the training cohort. **(B)** The decision curve of the C-A combined model for predicting the risk of poor prognosis of aSAH in the testing cohort.

A multi-predictor nomogram (incorporating age, the volume of SAH, ICH, and R-score) was created based on the C-A combined model to accurately assess the likelihood of a poor outcome in patients with aSAH (Figure 4). The DeLong test indicated that there was no statistically significant difference in the AUCs of the C-A combined model between the training and testing cohorts (*p* = 0.641). The calibration curve demonstrated that the C-A combined nomogram exhibited consistent predictive performance in both the training and testing groups (Figures 5A,B). DCA proved that the combined nomogram had good clinical benefits in the training and testing cohorts (Figures 6A,B).

## 4 Discussion

In this study, we used the radiomics characteristics of aneurysms in an innovative manner to predict the prognosis of patients with aSAH, and found that the R-score was an independent risk factor for poor outcomes. Among the three models developed, the C-A combined model had the best predictive performance and could effectively and reliably predict the short-term prognosis for patients with aSAH.

In our study, age, total volume of SAH, and ICH were proven to be independent risk factors for poor prognosis. Old age is considered a risk factor of adverse outcomes in patients affected by aSAH (Zheng et al., 2019; Rosengart et al., 2007). In agreement with this, we found that older patients are more likely to have a poor prognosis compared to young patients. This can be interpreted as an increase in the incidence of complications in case of older patients, which leads to a rapid deterioration of their condition. Similar to previous studies (Lagares et al., 2015; van der Steen et al., 2020; Boers et al., 2014), our data suggest that a larger volume of SAH is associated with poorer functional outcomes. Cerebral vasospasm produced by blood breakdown products in the subarachnoid space has been directly linked to the total volume of SAH. Vasospasms are closely associated with delayed cerebral ischemia and poor functional outcomes (Savarraj et al., 2021). In our study, ICH was also associated with adverse outcomes, which has been confirmed in a previous study (Rosengart et al., 2007) as well. This may be explained by the fact that a larger hematoma produces higher intracranial pressure, resulting in more severe early brain injury.

Radiomics features can objectively and comprehensively reflect some characteristics of the aneurysm that cannot be recognized by the naked eye. We extracted six radiomics features of aneurysms (The Maximum2D Diameter Column, cluster shade, IDMN, inverse variance, small area emphasis, and skewness). They provided a more comprehensive description of the aneurysm characteristics and were proven to be predictive markers of aSAH prognosis. The Maximum2D Diameter Column represents the aneurysm size. Although the influence of the aneurysm size on the prognosis of aSAH remains controversial (Russell et al., 2003; Liu et al., 2016; Roos et al., 2000), our results are consistent with those (Roos et al., 2000) suggesting that the larger the aneurysm, the higher the risk of poor prognosis. This is because vascular damage is more severe when larger intracranial aneurysms rupture, and the volume of the subarachnoid hemorrhage increases. Both these factors result in poor prognosis. In addition, larger aneurysms adhere more easily to other tissues and blood vessels, and are very difficult to expose and eliminate surgically. This is aslo likely to lead to a poor prognosis. Skewness describes the textural features of aneurysms from a microscopic perspective, and can better describe the shape of a ruptured IA. An irregular shape is a risk factor for SAH and results in increased subarachnoid blood loss and poor prognosis (Liu et al., 2016). Cluster shade, IDMN, inverse variance, and small area emphasis all reflecting the intensity level of voxel spatial distribution, were significantly different between good and poor outcomes.

In this study, the clinical model constructed using age, the volume of SAH, and ICH showed good predictive performance (AUC = 0.848) superior to that previously reported (Dengler et al., 2021) using Fisher Scale or modified Fisher Scale on admission (AUC = 0.55 and AUC = 0.65, respectively). This suggests that the use of quantitative data on admission can achieve a better predictive performance. With the inclusion of the R-score, age, the volume of SAH, and ICH, we develop our C-A combined model that demonstrated the best predictive power (AUC = 0.869). The accuracy and specificity of this model were higher than that of the clinical model, even if the DeLong test showed no statistical significance. In fact, in high-stakes situations, any incremental gain in predictive power could influence treatment strategies or patient management. Similarly, in a previous study (García-García et al., 2023), the limited improvement observed after the addition of clinical information suggested that many factors influencing patient outcomes are present in the early stages of the disease and could be identified from the initial CT scan. Therefore the C-A combined model featuring more independent predictors (especially the radiomics characteristics of the aneurysm itself) could improve forecasting performance. This type of prognostic models can provide help with clinical decisions at the initial stages and prevent the implementation of inadequate treatments, which is of utmost importance in emergency situations.

Finally, we developed a nomogram based on the C-A combined model that can be used to determine the total score according to the risk factors exhibited by patients on admission and thus obtain an early individualized assessment of the prognosis. In addition, the calibration curve of the C-A combined model showed a good calibration, indicating that the probability of poor outcome predicted by the model was in good agreement with the actual probability.

This study has nevertheless some limitations. First, our understanding of the mechanisms by which radiomic features influence prognosis is insufficient, and further studies are needed in the future. Second, the sample size was relatively small and this was a retrospective, cross-sectional study. In the future, we will strive to recruit more participants and conduct a longitudinal study to further optimize the prediction model. Third, although many variables were included in our study, not all the variables were covered. Therefore, further studies that consider a more comprehensive set of variables are warranted. Finally, to improve the applicability of our C-A combined model, a larger number of patients from multiple centers is needed.

In conclusion, age, total volume of SAH and ICH, and the R-score can predict the poor outcomes in patients affected by aSAH, and the nomogram based on the C-A combined model that takes them into account has been shown to be an accurate and practical tool for prediction. The model and the nomogram will hopefully help clinicians to identify patients at high risk of poor outcome on admission and optimize their treatment schedule.

## Data Availability

The original contributions presented in the study are included in the article, further inquiries can be directed to the corresponding authors.

## Footnotes

^1^https://www.shukun.net/

^2^http://www.itksnap.org/pmwiki/pmwiki.php

## References

[ref1] Boers A. M. Zijlstra I. A. Gathier C. S. van den Berg R. Slump C. H. Marquering H. A.. (2014). Automatic quantification of subarachnoid hemorrhage on noncontrast CT. AJNR Am. J. Neuroradiol. 35, 2279–2286. doi: 10.3174/ajnr.A4042, PMID: 25104292 PMC7965299

[ref2] Connolly E. S. Rabinstein A. A. Carhuapoma J. R. Derdeyn C. P. Dion J. Higashida R. T.. (2012). Guidelines for the management of aneurysmal subarachnoid hemorrhage: a guideline for healthcare professionals from the American Heart Association/American Stroke Association. Stroke 43, 1711–1737. doi: 10.1161/STR.0b013e3182587839 22556195

[ref3] Dengler N. F. Madai V. I. Unteroberdörster M. Zihni E. Brune S. C. Hilbert A.. (2021). Outcome prediction in aneurysmal subarachnoid hemorrhage: a comparison of machine learning methods and established clinico-radiological scores. Neurosurg. Rev. 44, 2837–2846. doi: 10.1007/s10143-020-01453-6, PMID: 33474607 PMC8490233

[ref4] Duan Z. Li Y. Guan S. Ma C. Han Y. Ren X.. (2018). Morphological parameters and anatomical locations associated with rupture status of small intracranial aneurysms. Sci. Rep. 8:6440. doi: 10.1038/s41598-018-24732-1, PMID: 29691446 PMC5915554

[ref5] Duan G. Yang P. Li Q. Zuo Q. Zhang L. Hong B.. (2016). Prognosis predicting score for endovascular treatment of aneurysmal subarachnoid hemorrhage: a risk modeling study for individual elderly patients. Medicine 95:e2686. doi: 10.1097/MD.0000000000002686, PMID: 26886607 PMC4998607

[ref6] García-García S. Cepeda S. Müller D. Mosteiro A. Torné R. Agudo S.. (2023). Mortality prediction of patients with subarachnoid hemorrhage using a deep learning model based on an initial brain CT scan. Brain Sci. 14:10. doi: 10.3390/brainsci14010010, PMID: 38248225 PMC10812955

[ref7] Hanley D. F. Lane K. Mcbee N. Ziai W. Tuhrim S. Lees K. R.. (2017). Thrombolytic removal of intraventricular haemorrhage in treatment of severe stroke: results of the randomised, multicentre, multiregion, placebo-controlled CLEAR III trial. Lancet 389, 603–611. doi: 10.1016/S0140-6736(16)32410-2, PMID: 28081952 PMC6108339

[ref8] Jiménez-Roldán L. Alén J. F. Gómez P. A. Lobato R. D. Ramos A. Munarriz P. M.. (2013). Volumetric analysis of subarachnoid hemorrhage: assessment of the reliability of two computerized methods and their comparison with other radiographic scales. J. Neurosurg. 118, 84–93. doi: 10.3171/2012.8.JNS12100, PMID: 22998059

[ref9] Konczalla J. Seifert V. Beck J. Güresir E. Vatter H. Raabe A.. (2018). Outcome after Hunt and Hess Grade V subarachnoid hemorrhage: a comparison of pre-coiling era (1980–1995) versus post-ISAT era (2005–2014). J. Neurosurg. 128, 100–110. doi: 10.3171/2016.8.JNS161075, PMID: 28298025

[ref10] Lagares A. Jiménez-Roldán L. Gomez P. A. Munarriz P. M. Castaño-León A. M. Cepeda S.. (2015). Prognostic value of the amount of bleeding after aneurysmal subarachnoid hemorrhage: a quantitative volumetric study. Neurosurgery 77, 898–907. doi: 10.1227/NEU.0000000000000927, PMID: 26308629

[ref11] Lambin P. Rios-Velazquez E. Leijenaar R. Carvalho S. van Stiphout R. G. P. M. Granton P.. (2012). Radiomics: extracting more information from medical images using advanced feature analysis. Eur. J. Cancer 48, 441–446. doi: 10.1016/j.ejca.2011.11.036, PMID: 22257792 PMC4533986

[ref12] Liu Q. Jiang P. Jiang Y. Ge H. Li S. Jin H.. (2019). Prediction of aneurysm stability using a machine learning model based on PyRadiomics-derived morphological features. Stroke 50, 2314–2321. doi: 10.1161/STROKEAHA.119.025777, PMID: 31288671

[ref13] Liu J. Song J. Zhao D. Li H. Lu Y. Wu G.. (2016). Risk factors responsible for the volume of hemorrhage in aneurysmal subarachnoid hemorrhage. Neurol. India 64, 686–691. doi: 10.4103/0028-3886.185398, PMID: 27381115

[ref14] Mackey J. Khoury J. C. Alwell K. Moomaw C. J. Kissela B. M. Flaherty M. L.. (2016). Stable incidence but declining case-fatality rates of subarachnoid hemorrhage in a population. Neurology 87, 2192–2197. doi: 10.1212/WNL.0000000000003353, PMID: 27770074 PMC5123555

[ref15] Parekh A. Satish S. Dulhanty L. Berzuini C. Patel H. (2023). Clinical prediction models for aneurysmal subarachnoid hemorrhage: a systematic review update. J. Neurointerv. Surg. jnis-2023-021107. doi: 10.1136/jnis-2023-021107, PMID: 38129109

[ref16] Petridis A. K. Kamp M. A. Cornelius J. F. Beez T. Beseoglu K. Turowski B.. (2017). Aneurysmal subarachnoid hemorrhage. Dtsch. Arztebl. Int. 114, 226–236. doi: 10.3238/arztebl.2017.0226 28434443 PMC5624452

[ref17] Rinkel G. J. E. Algra A. (2011). Long-term outcomes of patients with aneurysmal subarachnoid haemorrhage. Lancet Neurol. 10, 349–356. doi: 10.1016/S1474-4422(11)70017-5 21435599

[ref18] Roos E. J. Rinkel G. J. Velthuis B. K. Algra A. (2000). The relation between aneurysm size and outcome in patients with subarachnoid hemorrhage. Neurology 54, 2334–2336. doi: 10.1212/WNL.54.12.2334 10881266

[ref19] Rosengart A. J. Schultheiss K. E. Tolentino J. Macdonald R. L. (2007). Prognostic factors for outcome in patients with aneurysmal subarachnoid hemorrhage. Stroke 38, 2315–2321. doi: 10.1161/STROKEAHA.107.484360 17569871

[ref20] Russell S. M. Lin K. Hahn S. A. Jafar J. J. (2003). Smaller cerebral aneurysms producing more extensive subarachnoid hemorrhage following rupture: a radiological investigation and discussion of theoretical determinants. J. Neurosurg. 99, 248–253. doi: 10.3171/jns.2003.99.2.0248 12924696

[ref21] Savarraj J. P. J. Hergenroeder G. W. Zhu L. Chang T. Park S. Megjhani M.. (2021). Machine learning to predict delayed cerebral ischemia and outcomes in subarachnoid hemorrhage. Neurology 96, e553–e562. doi: 10.1212/WNL.0000000000011211, PMID: 33184232 PMC7905786

[ref22] van der Steen W. E. Marquering H. A. Ramos L. A. van den Berg R. Coert B. A. Boers A. M. M.. (2020). Prediction of outcome using quantified blood volume in aneurysmal SAH. AJNR Am. J. Neuroradiol. 41, 1015–1021. doi: 10.3174/ajnr.A6575, PMID: 32409315 PMC7342737

[ref23] Virani S. S. Alonso A. Benjamin E. J. Bittencourt M. S. Callaway C. W. Carson A. P.. (2020). Heart disease and stroke statistics-2020 update: a report from the American Heart Association. Circulation 141, e139–e596. doi: 10.1161/CIR.0000000000000757 31992061

[ref24] Wiebers D. O. Whisnant J. P. Huston J. 3rd Meissner I. Brown R. D. Jr. Piepgras D. G.. (2003). Unruptured intracranial aneurysms: natural history, clinical outcome, and risks of surgical and endovascular treatment. Lancet 362, 103–110. doi: 10.1016/S0140-6736(03)13860-3, PMID: 12867109

[ref25] Woo P. Y. M. Tse T. P. K. Chan R. S. K. Leung L. N. Y. Liu S. K. K. Leung A. Y. T.. (2017). Computed tomography interobserver agreement in the assessment of aneurysmal subarachnoid hemorrhage and predictors for clinical outcome. J. Neurointerv. Surg. 9, 1118–1124. doi: 10.1136/neurintsurg-2016-012576, PMID: 29030464

[ref26] Yang W.-S. Li Q. Li R. Liu Q.-J. Wang X.-C. Zhao L.-B.. (2018). Defining the optimal midline shift threshold to predict poor outcome in patients with supratentorial spontaneous intracerebral hemorrhage. Neurocrit. Care. 28, 314–321. doi: 10.1007/s12028-017-0483-7, PMID: 29139015

[ref27] Zheng Y. Xu F. Ren J. Xu Q. Liu Y. Tian Y.. (2016). Assessment of intracranial aneurysm rupture based on morphology parameters and anatomical locations. J. Neurointerv. Surg. 8, 1240–1246. doi: 10.1136/neurintsurg-2015-012112, PMID: 26863105

[ref28] Zheng K. Zhong M. Zhao B. Chen S.-Y. Tan X.-X. Li Z.-Q.. (2019). Poor-grade aneurysmal subarachnoid hemorrhage: risk factors affecting clinical outcomes in intracranial aneurysm patients in a multi-center study. Front. Neurol. 10:123. doi: 10.3389/fneur.2019.00123, PMID: 30873104 PMC6400833

[ref29] Zhu D. Chen Y. Zheng K. Chen C. Li Q. Zhou J.. (2021). Classifying ruptured middle cerebral artery aneurysms with a machine learning based, radiomics-morphological model: a multicentral study. Front. Neurosci. 15:721268. doi: 10.3389/fnins.2021.721268, PMID: 34456680 PMC8385786

